# Peripheral Skin Temperature and Circadian Biological Clock in Shift Nurses after a Day off

**DOI:** 10.3390/ijms17050623

**Published:** 2016-04-26

**Authors:** Massimo Bracci, Veronica Ciarapica, Alfredo Copertaro, Mariella Barbaresi, Nicola Manzella, Marco Tomasetti, Simona Gaetani, Federica Monaco, Monica Amati, Matteo Valentino, Venerando Rapisarda, Lory Santarelli

**Affiliations:** 1 Occupational Medicine, Department of Clinical and Molecular Sciences, Polytechnic University of Marche, Via Tronto 10/A, Ancona 60126, Italy; veronica.ciarapica@virgilio.it (V.C.); nicolamanzella@virgilio.it (N.M.); m.tomasetti@univpm.it (M.T.); simonagae87@hotmail.it (S.G.); monaco_federica@virgilio.it (F.M.); m.amati@univpm.it (M.A.); m.valentino@univpm.it (M.V.); l.santarelli@univpm.it (L.S.); 2 Healthcare Workers Service, ASUR Area 2, Loreto Hospital, Via S. Francesco 1, Loreto 60025, Italy; alfredo.copertaro@sanita.marche.it (A.C.); mariella.barbaresi@sanita.marche.it (M.B.); 3 Section of Occupational Medicine, Department of Internal Medicine and Systemic Diseases, University of Catania, Via Santa Sofia 78, Catania 95123, Italy; dottore.rapisarda@gmail.com

**Keywords:** skin temperature, circadian rhythm, cortisol, melatonin, PER2 gene, circadian clocks, light dark cycle, circadian dysregulation, occupational health, shift work

## Abstract

The circadian biological clock is essentially based on the light/dark cycle. Some people working with shift schedules cannot adjust their sleep/wake cycle to the light/dark cycle, and this may result in alterations of the circadian biological clock. This study explored the circadian biological clock of shift and daytime nurses using non-invasive methods. Peripheral skin temperature, cortisol and melatonin levels in saliva, and *Per2* expression in pubic hair follicle cells were investigated for 24 h after a day off. Significant differences were observed in peripheral skin temperature and cortisol levels between shift and daytime nurses. No differences in melatonin levels were obtained. *Per2* maximum values were significantly different between the two groups. Shift nurses exhibited lower circadian variations compared to daytime nurses, and this may indicate an adjustment of the circadian biological clock to continuous shift schedules. Non-invasive procedures, such as peripheral skin temperature measurement, determination of cortisol and melatonin in saliva, and analysis of clock genes in hair follicle cells, may be effective approaches to extensively study the circadian clock in shift workers.

## 1. Introduction

Many biological processes have a circadian rhythm. It is estimated that about 2% to 10% of the whole genome shows a circadian pattern of expression [1,2,3,4,5]. This physiological rhythmicity of the circadian biological clock is entrained by external stimuli such as light and temperature [6,7,8,9]. The need for continuous production in the industry sector, and continuous patient’s care in the health sector, suggest that many workers cannot adjust their sleep/wake cycle to the light/dark cycle [10,11,12]. This may result in alterations of their biological rhythms which, over time, can cause the development of diseases [13,14,15]. The circadian biological clock can be studied using parameters, such as body temperature, cortisol and melatonin secretion, and clock gene expression [16].

Changes in body temperature are the final effects of several physiological processes that result in the production of heat and successive dispersion through the skin. Thermal homeostasis of core temperature is regulated by the preoptic anterior hypothalamus; the circadian pattern of core temperature peaks in the afternoon and falls in the early hours of the morning during sleep [17,18]. The distal skin temperature rhythm has a pivotal role in the regulation of the core temperature rhythm and sleepiness, since heat loss from the extremities drives the core temperature circadian rhythm [19,20,21]. The peripheral temperature rises in the evening and nighttime causing heat dispersion, facilitating sleep and the decrease of core body temperature. In contrast, the peripheral temperature decreases in the daytime raising the core temperature [19,22,23,24,25]. In a subject living on a conventional day-oriented schedule, cortisol levels reach their minimal values early in the night and reach their maximal values around the regular time of awakening [26,27,28]. Cortisol in blood is bound up with serum protein and this affinity is temperature-dependent [29,30]. Melatonin secretion starts in the evening, levels peak in the middle of the night, and slowly decline thereafter to reach their lowest levels at the end of the morning [31]. Melatonin secretion is inhibited by exposure to light at short wavelengths, a deregulation in its secretion was noted in many disease processes, including cancer [32,33]. Shift workers experience light exposure and sleep deprivation during night shifts, which results in reduced levels of melatonin [15,34].

Clock genes are expressed in both the hypothalamic suprachiasmatic nucleus and in other mammalian tissues where they regulate tissue-specific gene expression [35]. Clock gene expression is self-maintained by two transcriptional activators, namely CLOCK (circadian locomoter output cycles kaput) and BMAL1 (brain and muscle ARNT-like protein 1), which activate the expression of clock genes Period (*Per*) and Cryptochrome (*Cry*). PER and CRY repress the CLOCK-BMAL1 dimer resulting in negative feedback on the transcription of *Per* and *Cry*, thus closing the cycle in approximately 24 h [36,37,38,39]. Among clock genes, *Per2* expression is influenced by temperature and plays a role in the adaptation to cold [8,40,41].

In this study, we investigated peripheral skin temperature, cortisol and melatonin levels, and *Per2* expression in shift and daytime nurses using non-invasive methods.

## 2. Results

No significant differences in demographic characteristics, Epworth scores, and Chronotype (MEQ score) were observed between shift-working (SW) and daytime (DT) nurses (Table 1).

Wrist skin temperature showed a circadian rhythm in both SW and DT nurses (ANOVA repeated measures and Cosinor analysis, *p* < 0.05). Wrist skin temperatures of SW nurses had a lower mesor, and a lower amplitude and higher minimum compared to DT nurses; there was no difference between maximum values (Table 2).

**Figure 1:**
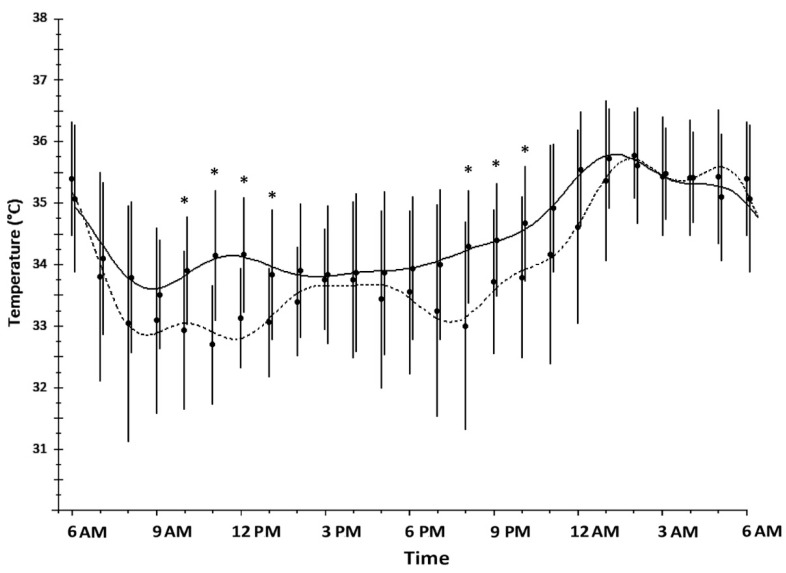
Profiles of wrist skin temperature of shift-working (solid line) and daytime (dashed line) nurses collected for a 24 h period. Data are expressed as the hourly mean ± SD. Statistical significance is indicated by * *p* < 0.05.

Wrist skin temperatures of SW nurses were significantly higher in the morning hours (from 10:00 AM to 1:00 PM) and in the evening (from 8:00 PM to 10:00 PM) (Figure 1).

**Figure 2:**
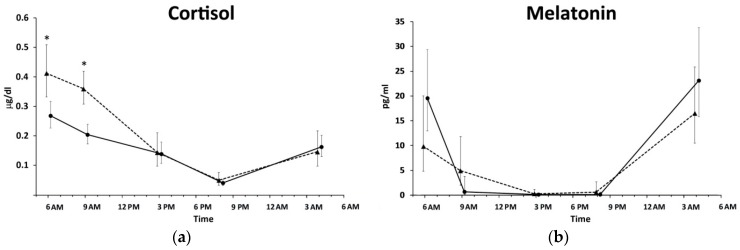
Profiles of cortisol (**a**) and melatonin (**b**) levels in saliva samples of shift-working (solid line) and daytime (dashed line) nurses collected for a 24 h period. Data are expressed as the geometric mean ±95% confidence interval. Statistical significance is indicated by * *p* < 0.05.

Cortisol levels were also significantly different in the 24 h period of both groups (ANOVA repeated measures, *p* < 0.05) with maximum values at 6:00 AM. Significant differences were found between SW and DT nurses in cortisol levels (ANOVA repeated measures, *p* < 0.05); cortisol levels were significantly lower in SW nurses than those of DT nurses at 6:00 AM and 8:00 AM (Figure 2).

Melatonin levels showed significant differences in the 24 h period of both DT and SW nurses (ANOVA repeated measures, *p* < 0.05); no significant differences were found between the two groups (Figure 2). Maximum levels were noted at 4:00 AM in both groups but no significant difference was found between the two groups.

**Figure 3:**
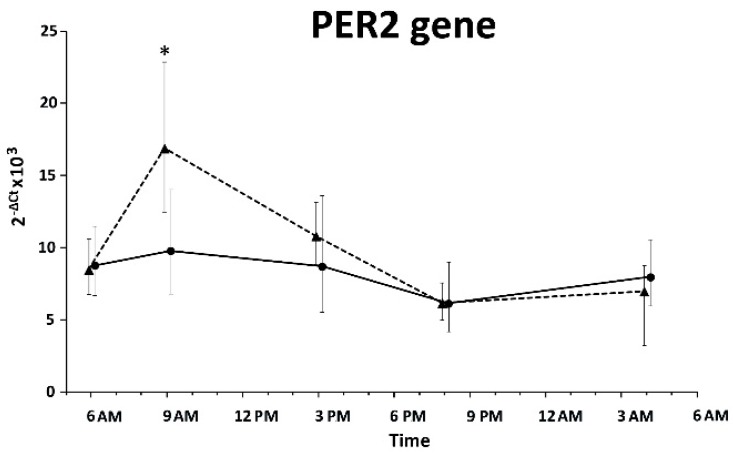
Profiles of *Per2* expression in pubic hair follicle cells of shift-working (solid line) and daytime (dashed line) nurses collected for a 24 h period. Data are expressed as the geometric mean ± 95% confidence interval. Statistical significance is indicated by * *p* < 0.05.

Samples collected from 20 SW and 22 DT nurses were tested for *Per2* expression in pubic hair follicle cells. Sufficient amounts of mRNA were not obtained from six subjects. The expression of *Per2* was significantly different in the 24 h period of both SW and DT nurses, the 24 h variations were less significant in SW nurses (ANOVA repeated measures, *p* < 0.05) (Figure 3). The expression of *Per2* was statistically no different between the two groups except in the maximum levels at 8:00 AM which were significantly lower in SW nurses.

## 3. Discussion

The circadian biological clock is primarily regulated by a light/dark cycle that is naturally based on sunlight. This cycle has caused humans to sleep during nighttime. The biological clock ensures both efficiency and energy saving in several physiologic processes of subjects living on a conventional day-oriented schedule. Shift work, involving night work, may desynchronize circadian rhythms causing persistent mismatching between the sleep/wake cycle and the light/dark cycle [15,42]. Workers in the study showed alterations in peripheral skin temperature. The peripheral skin temperature is found to have a higher mesor in SW nurses, the maximum is not different while the minimum is significantly greater, and consequently the amplitude is smaller. A higher wrist skin temperature was observed in SW nurses from 10:00 AM to 1:00 PM suggesting a minor ergotropic activation confirmed by the low levels of cortisol in the morning. We anticipated a higher diurnal sleepiness in SW, but the hypothesis was not confirmed by the Epworth score, which was similar between groups. A higher wrist skin temperature was observed also from 8:00 PM to 10:00 PM suggesting an anticipated propensity to sleep. SW nurses did not show the wrist skin temperature increase in the early afternoon present in DT; this phenomenon was observed in obese women, since alterations in peripheral temperature were found to be associated with metabolic syndrome [43,44]. No difference in Body Mass Index (BMI) was observed in our sample. However, it is not possible to exclude the possibility that wrist skin temperature rhythm alteration may be preliminary to a development of metabolic syndrome associated with shift-work [45,46].

Light exposure influences thermoregulation [9,47]. Nonphysiological light/dark cycles caused by night schedule may influence a thermophysiological response, as well as melatonin secretion [6,9]. Melatonin has thermoregulatory action both as a vascular modulator and as a molecule related to the amount and functionality of brown adipose tissue [48]. Alteration in melatonin secretion during the night shift may not persist after a day off [34,49]. The melatonin alteration is strongly influenced by the number of night shifts, specific work schedule, and light intensity exposure during night shifts [50,51,52,53,54]. In this study, the SW nurses did not show significant differences from DT nurses in melatonin levels. Since actual peaks may have been earlier than 4:00 AM, differences in the acrophase of melatonin may be present but not captured; results might also be affected by the night’s sleep of SW nurses the day before sampling. However, taking into consideration the low number (mean = 6.0) of participant’s night shifts per month in this study, results are in line with a previous study conducted where a decrease of melatonin was only found to be associated with ≥8 night shifts per month [53].

SW showed reduced cortisol levels in the morning. Typically, cortisol secretion increases at 6:00 AM and decreases at 9:00 PM [55]. Fluctuations in the cortisol profiles are lower during night shifts than during the day shifts [56,57]. Resumption of the regular cortisol secretion was observed in SW after one or more days off [56,58]. Our results support theories that 48 h of rest is not sufficient to SW to restore cortisol levels similar to DT nurses. The cortisol levels after awakening, and diurnal changes, can be used to indicate the adjustment required to meet environmental challenges [59]. Cortisol levels are the expression of the central circadian pacemaker and they represent a key molecule used to transfer circadian information from brain to peripheral tissue [60]. Glucocorthicoids stimulate clock gene expression [61,62], thus *Per2* expression in hair follicle cells is probably modulated by cortisol levels. It could be interesting to study other clock gene expression levels in SW hair follicle cells, since clock genes as *Per1* and *Per3* were found to have robust circadian rhythms in beard follicle cells [63]. Few studies until now investigated clock genes in SW with different results [34,49,64,65,66]. Peripheral levels of clock genes expression fluctuate naturally in a circadian fashion, and they can be influenced by a number of internal and external cues [61,67,68]. Hair follicle cells permit the study of clock gene expression in multiple time samplings limiting the misinterpretation of data from a single time point.

A desynchronization of the sleep/wake cycle with the light/dark cycle is typical of SW. This may result in alterations of central and peripheral homeostatic mechanisms such as thermoregulation. The alterations observed in the SW nurses are not necessarily detrimental to health since they may be a physiologic adaptation to continuous circadian desynchronization. The necessity of achieving homeostasis is obtained with a lower investment in circadian variations during daytime. This energy saving strategy might lead, in the long term, to alterations related to metabolic syndrome.

Non-invasive procedures, such as peripheral skin temperature measurement, determinations of cortisol and melatonin in saliva, and the analysis of clock genes in hair follicle cells, may be effective approaches to extensively study the circadian biological clock in SW.

## 4. Experimental Section

### 4.1. Participants and Sampling

Participants were female nurses of the National Health Service hospital wards in Ancona (Italy). SW nurses were employed in a “clockwise rapidly rotating” type of shift-work. The schedule was as follows: Day 1: 7:00 AM–2:00 PM; Day 2: 2:00 PM–10:00 PM; Day 3: 10:00 PM–7:00 AM; 48 h of rest; resumption of the cycle. The work schedule of DT nurses was from 7:00 AM to 2:00 PM six days/week. The study was carried out in accordance with the Declaration of Helsinki’s ethical standards. As part of the standard occupational health surveillance, the study needed no formal approval by the local ethics committee. Nevertheless, the committee was consulted and it granted an informal authorization. Nurses were evaluated and selected based on the following criteria investigated during medical examination: fertile age (presence of a menstrual cycle), no current treatment with drugs, a negative history of psychiatric disorders, degenerative or cardiovascular diseases, insomnia, chronic viral infections, tumor or autoimmune diseases, no skin pathologies, no occupational exposure to ionizing radiation or involvement in antiblastic drug preparation, absence of artificial light (no light sources in the bedroom, no light from windows) when sleeping at home. Additional selection criteria were taken into account for SW and DT nurses. SW nurses had to be assigned to the current shift schedule involving at least 60 night-shifts/year without schedule breaks in the previous six6 months for at least two years. DT nurses had to possess a habitual sleep/wake schedule approximately between the hours of 11:00 PM and 6:00 AM without an episode of sleep deprivation for at least three weeks prior to the study. Among 65 nurses meeting the selection criteria, 23 SW nurses and 25 DT nurses agreed to participate in the study and gave their written informed consent. For both SW and DT nurses, biological samples were collected on the working day after a day off. In this working day, both groups had a morning-shift thus they were as comparable as possible preventing the acute alterations in SW nurses due to light at night exposure and sleep deprivation associated with the night-shift.

Participants filled in a questionnaire enquiring into their work schedule and other habits, such as smoking and alcohol consumption. The Epworth Sleepiness Scale was used to assess daytime sleepiness [69,70]. The chronotype was assessed by the “Morningness-Eveningness Questionnaire” (MEQ) [71], a 19-item questionnaire with a total score ranging from 16 to 86, extensively used in adults and workers [72,73,74]. All nurses had to participate in a tutorial about the procedure of sampling and successive conservation. Biological samples (saliva and pubic hair) were self-collected by nurses at 6:00 AM, 9:00 AM, 3:00 PM, 8:00 PM, and 4:00 AM of the successive day. Nurses conserved the biological samples at a temperature ranging from 2 to 8 °C, a thermal bag was used to transport samples. The temperature of conservation was continuously registered by a temperature data logger (Thermochron iBotton DS1922L, Maxim Integrated Products, Inc., Sunnyvale, CA, USA) fixed to the specimen containers. All samples were delivered to the laboratory the morning after the last sampling.

### 4.2. Peripheral Skin Temperature Measurement

Peripheral skin temperature was assessed continuously at the wrist on the nondominant hand as described by Sarabia *et al*. [75]. Temperature measurements were done by a Thermochron iButton DS1922L (Maxim Integrated Products, Inc., Sunnyvale, CA, USA). The use of iButtons for human skin temperature measurement has been noted by some authors [76,77,78]. The accuracy is −0.09 °C with a precision of 0.05 °C [77]. For the DS1922L, manufacturer’s specifications state this latest model functions throughout the full human thermo-physiological range. For this study, the iButton resolution was set at 0.0625 °C and the iButton real-time clock was synchronized with that of a laptop computer. The sensor was programmed to sample every 2 min for 24 h starting at 6:00 AM on the first working day after a day off. Wrist skin temperature was investigated the week before the sampling of the biological material in order to avoid artifacts of the wake-up for sampling at 4:00 AM. An iButton was attached to a cotton wrist band and the sensor surface was placed over the inside of the wrist on the radial artery. The data stored in the temperature sensor were transferred through an adapter (Thermochron iBotton DS9490B, Maxim Integrated Products, Inc., Sunnyvale, CA, USA) to a personal computer using OneWireViewer v. 3.15.50 (Maxim Integrated Products, Inc., Sunnyvale, CA, USA).

### 4.3. Cortisol and Melatonin Measurement

Saliva is an ideal matrix for multiple non-invasive sampling and allows the study of hormones such as cortisol and melatonin. Salivary cortisol levels are unaffected by salivary flow rate and are relatively resistant to degradation from enzymes or freeze-thaw cycles [79,80]. Studies reported that serum and salivary cortisol are strongly correlated, indicating that salivary cortisol levels reliably estimate serum cortisol levels [81,82]. Melatonin levels in plasma are paralleled by corresponding variations in saliva where the salivary concentration is about 30% of that found in plasma. Measurement of salivary melatonin is advantageous especially to avoid invasive venipuncture procedures [83,84]. Saliva samples were collected by unstimulated passive drool, passing the saliva directly into a polypropylene tube. Upon arrival at the laboratory, saliva samples were stored at −80 °C until required for assay. On the day of assay, the samples were thawed for approximately 4 h until room temperature (20.0–23.0 °C). Samples were then centrifuged at 3000 rpm for 15 min to remove mucins and other particulate matter which may interfere with analysis.

The free cortisol levels in saliva were determined in duplicate using a high-sensitivity salivary cortisol enzyme immunoassay kit (Salimetrics, LLC, State College, PA, USA) according to the manufacturer’s instructions. Samples from each subject were assayed in the same batch. The inter- and intra-assay variations were below 6.2% and 3.9%, respectively.

The melatonin levels were determined in duplicate using a salivary melatonin enzyme immunoassay kit (Salimetrics, LLC, State College, PA, USA) according to the manufacturer’s instructions. Samples from each subject were assayed in the same batch. The inter- and intra-assay variations were below 8.9% and 5.4%, respectively.

### 4.4. Circadian Expression of PER2 Clock Gene

The circadian expression of peripheral clock genes can be found in the blood and in other peripheral tissues [85,86,87]. Multiple blood sampling constitutes a major limit of studies on clock genes involving free-living subjects. This problem may be bypassed using hair follicle cells that constitute suitable biological samples for clock genes determination [63,88]. Total RNA quality and quantity in follicle cells were preliminarily tested in hairs collected from scalp, eyebrows, arms, pubic region, and legs of female volunteers. Hairs collected from the pubic region were selected for the study as they provided the best quality and quantity of total RNA. In each sampling, nurses harvested 15 pubic hairs. They were immediately submerged into tubes containing a RNA stabilization reagent (RNA Later, Sigma, Saint Louis, MO, USA).

When samples were delivered to the laboratory, RNA extraction was performed immediately. The isolation of total RNA was performed using the RNeasy Mini Kit (QIAGEN, Hilden, Germany) according to the manufacturer’s instructions. Obtained RNA was concentrated by Eppendorf Concentrator 5301 (Eppendorf, Hamburg, Germany). RNA quality and quantification were evaluated with a Nanodrop 1000 spectrophotometer (Thermo Scientific, Wilmington, DE, USA). cDNA was synthesized according to the of High-Capacity cDNA Reverse Transcription Kit protocol (Applied Biosystems, Foster City, CA, USA). Gene expression was analyzed by real-time quantitative PCR using the TaqMan Gene Expression Master Mix (Applied Biosystems, Foster City, CA, USA). To control for variation in the amount of cDNA available for PCR in the different samples, the gene expression levels of the target sequences were normalized to the expression of an endogenous control, glyceraldehyde-3-phosphate dehydrogenase (*Gapdh*). Primer and probe sets used for RT-PCR assays were as follows: Hs.PT.58.3464649 for *Per2* and Hs.PT.39a.22214836 for *Gapdh* (Integrated DNA Technologies Inc., Coralville, IA, USA). The expression levels of *Per2* was calculated applying the following equation: 2^−∆*C*t^.

### 4.5. Statistical Analysis

The normality distribution of variables was assessed by the Kolmogorov-Smirnov test. Natural logarithms of cortisol, melatonin, and *Per2* expression values were used for analysis as transformed values more closely approximated a normal distribution. Continuous variables were expressed as mean and standard deviation, while log-transformed variables were expressed as a geometric mean along with a 95% confidence interval (95% CI). Repeated-measures of ANOVA were performed to analyze between- and within-groups a change of variables across time. The Mauchly test was performed to verify the sphericity assumption. When statistical differences were found by repeated measures of ANOVA, a multiple-comparison test, adjusted by the least significant difference, was applied to identify the differences between the two groups for each time point. Wrist skin temperature was further analyzed by Cosinor analysis, calculating its mesor, amplitude, acrophase, and maximum and minimum values. Student’s *t*-test was used to test differences of independent variables, and maximum and minimum values, of each variable between the two groups. Data was analyzed by Statistical Package Social Sciences (version 19) software (SPSS, Chicago, IL, USA) and Chronos-Fit 1.06 [89].

## Figures and Tables

**Table 1:** Demographic characteristics, Epworth Sleepiness Scale scores, and Chronotype (MEQ score) of shift-working (SW) and daytime (DT) nurses.

Parameters	SW Nurses (*n* = 23)	DT Nurses (*n* = 25)	*p*-Value
Age (years) mean ± SD	38.8 ± 3.9	39.2 ± 3.2	0.699
Job seniority (years) mean ± SD	13.6 ± 3.4	12.8 ± 4.4	0.487
Shift-work seniority (years) mean ± SD	13.6 ± 3.4	-	-
Night-shift work (nights per month) mean ± SD	6.0 ± 1.0	-	-
Body Mass Index (kg/m^2^) mean ± SD	24.2 ± 4.7	25.0 ± 4.3	0.541
Smokers (%)	39.1	44.0	0.732
Alcohol drinkers (%)	34.8	40.0	0.709
Epworth Sleepiness Scale (score) mean ± SD	6.0 ± 3.7	5.9 ± 3.1	0.919
Chronotype ^a^ (MEQ score) mean ± SD	54.3 ± 7.8	57.2 ± 9.6	0.259

^a^ An higher score is indicative of morningness preference.

**Table 2:** Results of Cosinor analysis of wrist skin temperature of shift-working (SW) and daytime (DT) nurses.

Wrist Skin Temperature	SW Nurses (*n* = 23)	DT Nurses (*n* = 25)	*p*-Value
Mesor (°C) mean ± SD	34.47 ± 0.42	33.96 ± 0.62	<0.001
Maximum (°C) mean ± SD	36.20 ± 0.45	36.31 ± 0.46	0.407
Minimum (°C) mean ± SD	32.85 ± 0.92	31.59 ± 0.99	<0.001
Amplitude (°C) mean ± SD	0.95 ± 0.44	1.23 ± 0.38	0.022
Acrophase (h) mean ± SD	5:05 ± 7:43	4:05 ± 5:50	0.617
